# Perceptions and Experiences of Professional Nurse Educators and Midwives on Simulation-Based Education in Tanzania: A Qualitative Study

**DOI:** 10.3390/healthcare14080994

**Published:** 2026-04-10

**Authors:** Paulo Lino Kidayi, Christina Chuck Mtuya, Eva-Christina Risa, Jane Januarius Rogathi

**Affiliations:** 1 School of Nursing, KCMC University, Kilimanjaro, Moshi P.O. Box 2240, Tanzania; christina.mtuya@kcmcu.ac.tz (C.C.M.); jane.rogathi@kcmcu.ac.tz (J.J.R.); 2 Faculty of Health Sciences, University of Stavanger, P.O. Box 8600, Forus, 4036 Stavanger, Norway; christina.f.risa@uis.no

**Keywords:** simulation, education, knowledge, perception, experience, nurse educators, midwife, graduates

## Abstract

**Background:** Evidence shows that simulation-based education for nurses and midwives contributes to strengthening patient safety and quality of care in healthcare settings. Nevertheless, it is implemented to a limited degree in Sub-Saharan African (SSA) higher education institutions, including Tanzania. This demands that Tanzania shift from a traditional model of teaching to incorporate simulation-based education to produce a skilled workforce. **Objective**: To explore perceptions and experiences of nurse educators (lecturers) and midwives on simulation-based education in Tanzania. **Methods:** The study employed a generic qualitative descriptive study design with purposive sampling. The data were collected through individual semi-structured interview guides with nurse educators and midwives (nine nurse educators and 11 midwife graduates) from two selected universities in the School of Nursing and their respective teaching hospitals. Qualitative inductive content analysis was used to analyze the data. **Results:** The data analysis revealed three themes and nine sub-themes: 1. Knowledge and skills in simulation-based education. 2. Challenges in the implementation of simulation-based education. 3. Ensuring patients’ safety. **Conclusions:** Students were indeed experienced, but not trained in how to use simulation-based education, and nurse educators had inadequate skills. A high number of students with inadequate infrastructure and resources is the major challenge experienced by participants. Simulation-based education is at an early stage of adoption in Tanzania and will require ongoing development, support and resources to fulfilll its potential in promoting patient safety.

## 1. Introduction

The simulation-based education (SBE) approach is evident in high-income countries, with minimal utilization in low-income countries [1,2]. SBE in healthcare is a pedagogical method that sets up a scenario or environment to allow people to experience a representation of a real healthcare event for learning [3]. Evidence shows that SBE as a pedagogic method is widely used for training healthcare providers and students, useful in supporting student learning of clinical decision-making [4]. Moreover, SBE demonstrated evidence to support its efficacy in academic and clinical contexts, to improve healthcare provision and outcomes through effective clinical judgment among nursing students for patient safety and quality of care [5,6,7].

In higher education programs, it has been found that simulation practice opportunities for problem-solving have an impact. This has been reported to facilitate the acquisition of targeted skills [6,8]. Student nurses’ experiences in SBE demonstrated positive motivation in caring for patients; they gained competence in teamwork, confidence, and communication skills [9,10]. SBE has been found to enhance clinical competence at the undergraduate and postgraduate levels [11].

In response to the great shortage of health workers in Tanzania [12,13], there has been a remarkable increase in the number of healthcare students enrolled in universities, including nursing students [14,15]. However, the expansion of the enrolment rate does not match with teaching staff, relevant clinical settings, and infrastructure in various universities [16]. For example, in the Northern zone, according to the university’s strategic plan 2021–2025, there was a 20% shortage of academic staff, including nurse educators. This could limit the capacity of training institutions to produce competent graduates, including midwives [17]. Moreover, graduates will not satisfy the demand of service consumers in a national and international context in promoting patient safety and quality of life. There is a need for transformative teaching strategies in Sub-Saharan Africa (SSA), including Tanzania [17,18], as the student population increases. The congestion of students [19] affects both nurse educators, clinical staff and enrolled students to fulfill learners’ competencies stipulated in the curriculum; thus, diverse teaching strategies are of paramount importance. Simulation skill training enhances confidence among learners before being exposed to the clinical setting [20], though this might be affected by the student–teacher ratio [21], and infrastructures in simulation laboratory and clinical settings.

This demands higher learning institutions to select effective pedagogic methods integrating SBE to promote patient safety. Pedagogy is the art and science of education, seeking to understand practices and methods of instruction that can help teachers educate learners [22]. SBE bridges the gap between theory and clinical practice, facilitating skill learning and promoting patient safety [23,24]. SBE demands well-established infrastructure and competent facilitators for the learner to gain the required skills [25]. Furthermore, the shift from a knowledge-based curriculum to a competence-based curriculum in higher learning institutions in Tanzania demands the integration of SBE as a pedagogic approach [25] to enhance practical skills.

In Tanzania, the nursing and midwifery curriculum was last reviewed in 2017 to ensure compliance with education standards. Three universities in Tanzania are using the same harmonized competence-based curriculum, including two universities that participated in the study. The simulation was at this point itemized as a teaching method [26] as opposed to more traditional teaching methods such as auditorium lectures and self-studies. Still, few studies are available from Tanzania on SBE in nursing education institution settings. Despite a high number of students with limited infrastructure, SSA, including Tanzania, is lagging in utilizing SBE, though speculation of the specific context is paramount. Evidence to support studies on the perception and experiences of nursing and midwifery postgraduate graduates on SBE, as well as nursing educators (lecturers), is minimal. The understanding of SBE and its utilization both in classroom teaching and simulation laboratory and clinical settings (hospitals) is vital across all institutions to contribute to the existing body of knowledge.

Therefore, the purpose of this research is to explore the perceptions and experiences of nurse educators (lecturers) and midwives to gain insight into the adoption of SBE in Tanzanian nurse education.

## 2. Materials and Methods

### 2.1. Research Question

What are the perceptions and experiences of nurse educators (lecturers) and midwives on simulation-based education in nursing and midwifery education programs in Tanzania?

### 2.2. Study Design

The study employed a generic qualitative descriptive study design [27] with purposive sampling and inductive content analysis [28,29] to explore perceptions and experiences of nurse educators and midwives on simulation-based education in Tanzania. A generic qualitative approach merely entails research that is not guided by explicit or established philosophic assumptions in the form of one of the known qualitative methodologies such as the big three, i.e., phenomenology, grounded theory and ethnography [27]. It focuses on understanding participants’ subjective experiences and perceptions of situations, and is thus appropriate for this group.

### 2.3. Study Setting

The study was carried out in two nursing and midwifery training institutions in the selected universities in the School of Nursing and their respective teaching hospitals in the Eastern and Northern zones in Tanzania. According to the Tanzania regulatory authority, each medical school university has a teaching hospital for clinical placement where students practice their skills and provide superspecialists healthcare services to the Tanzanian population. In the Eastern zone, the university and respective teaching hospitals are owned by the government, using SBE as a pedagogical approach both in a simulation laboratory and in the clinical setting. They had simulation preceptor corners and manikins in some of the departments, like the labor ward, Obstetrics and Gynaecology, and reproductive clinics. On the other hand, in the Northern zone, the university and its respective teaching hospital are faith-based institutions owned by the Good Samaritan Foundation in Tanzania, which were not implementing SBE. The graduates from Northern zone university were oriented on SBE whilst continuing professional development education from visiting lecturers at the university and in the clinical setting. Both institutions possess simulation laboratories for students to practice before being exposed to clinical placement.

### 2.4. Study Participants

The study comprised nurse educators (lecturers) and midwifery graduates. Midwife graduates are former students of a Master of Science in the midwifery postgraduate program, who graduated from the two selected universities and are working in the teaching hospitals, while nurse educators are experienced academicians employed by the universities and working as lecturers. This cohort was selected since postgraduate graduates are engaged in facilitating learning in clinical settings, and universities use the same curriculum. The total number of informants was 20 participants: 9 nurse educators and 11 midwife graduates agreed to participate in the study. This study included participants from two universities and their respective teaching hospitals. Two had a PhD from an abroad university and the rest had an MSc in midwifery (two year course).

#### 2.4.1. Inclusion Criteria

Nurse educators (lecturers) and midwife graduates working in the selected universities and their respective teaching hospitals for at least a minimum of six months were eligible to participate in the study. Males and females, aged 18 years and above, were included in a study.

#### 2.4.2. Exclusion Criteria

Nurse educators and midwives who were on annual or sick leave were excluded from the study.

Individuals with temporary university and teaching hospital appointments of less than six months, such as those on short-term contracts, were excluded from the study.

#### 2.4.3. Sampling Procedure

Purposive sampling [30] was employed in the study. This approach was employed as the group (nurse educators and midwife graduates) had ample information on the phenomena under study. The participant group was selected as they were regarded as being knowledgeable and experienced on the topic under study [31,32]. Varied gender and age groups among the participants participated in the study. Moreover, the two universities selected use one harmonized competency-based curriculum across the institutions, and both academic staff and midwife graduates were involved in curriculum development.

#### 2.4.4. Recruitment Procedure

The Dean’s School of Nursing at the university and heads of departments in the teaching hospitals were the first to contact people to meet with the researcher upon getting permission from respective authorities, who then introduced them to the participants. The researcher asked permission from the participants to be included in the study. Upon participant acceptance, the researcher explained the purpose, benefits and risks of the study to participants so that they were informed before agreeing to participate. Then, they were asked to sign a written informed consent form. The researcher assured informants that participation was voluntary, and the information would remain within the context of the study. Moreover, at any time, participants could withdraw from the study without losing any benefits.

### 2.5. Data Collection Tool and Methods

Data was collected through face-to-face interviews, and a semi-structured interview guide was used for data collection. The interview guide was developed by researchers for this study and comprised three sections: sociodemographic characteristics, knowledge on simulation-based education, and experiences on the use of simulation-based education. The interview guide was pre-tested on two participants to test the credibility of the tool before being used [30]. There was no adjustment of the tool after the pilot, and it remained the same throughout the period of data collection. Moreover, data from pre-test was not included in the study. Since the language of instruction in Tanzania, from secondary education to postgraduate, is English, the English version of the interview guide was used for data collection. The data collection was done in June 2023. Data collection was conducted by the second author. The interviews were conducted with all informants in a private room by the second author, a female postgraduate social scientist with formal training and experience in qualitative studies, including in-depth interviews. Reflexivity was achieved as, at the time of the study, the second author was affiliated with KCMC University and shared a similar cultural background with the participants, which facilitated rapport and open communication. There was no prior relationship between the interviewer and the participants before recruitment. Before agreeing to participate, participants were informed about the interviewer’s professional experience, institutional affiliation, and the aim of the study. The research team included specialists with backgrounds in nursing, midwifery, sociology, public health, epidemiology, and qualitative research. However, all researchers were conscious of and actively acknowledged their own beliefs, biases, and judgment systems regarding simulation-based education and training institutions. Throughout the study, the research team engaged in reflective discussions to identify and minimize potential biases based on their professional responsibilities and prior views about simulation-based education and training institutions.

The researchers (two female and one male) were trained on the research project protocol, including data collection. Since the research included two informants, the interview took place at each informant’s place. For nurse educators, the interview was conducted in a private room within their schools, while for midwives, it was conducted in a private room in the labor ward, and the interview lasted for 45–60 min. The interviews were audio-recorded with the permission of the participants, and field notes were taken. Data saturation was reached at the 18th interview. In this study, saturation was operationally defined as the point at which no new information emerged from the data during the ongoing process of data collection. By the time the 18th interview was analyzed, the data began to show strong thematic redundancy, with participants repeating similar experiences and perspectives already captured in earlier interviews. The 19th and 20th interviews were conducted to confirm this pattern, and no new information emerged. At this stage, only repetition and elaboration of existing information were observed. Therefore, the 18th interview confirmed that thematic saturation had been achieved, and further data collection was considered unlikely to yield substantially new information relevant to the study objectives [33,34]. However, two (2) participants were added to see if new information could emerge, though none appeared different from the previous respondents.

### 2.6. Data Analysis

Qualitative content analysis was used to analyze the data [35]. Data from the nurse educators (lecturers) and midwife graduates were analyzed together, though indicated in separate quotes. Data was transcribed verbatim [28], and the first author did the transcription. The second and last authors also listened to the audio to countercheck the originality of the transcribed data to ensure it retained its meaning. Re-reading was carried out to acquaint the overall concept of the content. The data material was shared through a protected data storage backup (external hard disk) since all the researchers were within the same working place. The first and second authors were involved in data analysis, and the process involved breaking down data into smaller units, coding, and naming the units according to the content in the text. The transcripts were coded independently by the first and second authors, and then co-coding was done by the same authors. Finally, face-to-face discussions were held to ensure all codes were performed similarly. The theme and subthemes emerged from the participants’ information after reaching consensus on the discussion between the first and second authors. The original meaning of the data was retained throughout the data analysis. This is a process of rendering participants’ original information. The authors finally reached a consensus on three main themes with a total of nine sub-themes. The three main themes that emerged were: (1) knowledge and skills in SBE, (2) challenges in the implementation of SBE, and (3) ensuring patients’ safety. To reduce the researcher’s repercussions during data analysis [36,37] in the qualitative approach, four criteria were applied: credibility, dependability, confirmability, and transferability.

Credibility was achieved using multiple analyses of the data, of which more than one author conducted co-coding, and the researchers’ team consensus on the emerging sub-themes and main themes revealed a rigorous approach to the emerging findings. Moreover, the study was conducted from two different study settings with varied informants, nurse educators, and nurse midwives with varied gender and age groups, which brought rich information into the broader context of the study. The interview guide was validated and pretested to ensure its reliability, and one researcher conducted the interview using the same tool, which ensured the quality of information gathered, thus enhancing dependability. The process of member checking during the process of data analysis and agreement reached in the emerged themes ensured the information derived from the participants’ original information, and thus confirmability was reached. The description of the research process context that gives the reader a clear view of the research findings guarantees the transferability of the information to a similar group and setting to replicate the context. The study reported the findings according to the COREQ checklist, which is used to report qualitative studies [38].

## 3. Results

### 3.1. Social Demographic Characteristics

This study included informants from two universities and their respective teaching hospitals. Most of the participants were female 18 (98%). Two had a PhD from an abroad university and the rest had an MSc in midwifery (two year course), as shown in Table 1.

### 3.2. Themes and Sub-Themes

Three main themes and nine sub-themes emerged from the analyses. The main themes identified were: (1) knowledge and skills in SBE, (2) challenges in the implementation of SBE, (3) ensuring patients’ safety. The main themes and sub-themes are presented in Table 2.

#### 3.2.1. Theme: Knowledge and Skills in SBE

The understanding of SBE and its utilization both in classroom teaching and in simulation laboratory and clinical settings (hospitals) is described according to the informants’ expressions. This theme illustrates how both nurse educators and midwife graduates perceived and experienced awareness, knowledge, skills and practice on SBE with other pedagogical approaches within the institutions.

1. Sub-theme: Lecturers and midwives’ awareness of SBE

Most of the informants had heard about SBE, and some had never received any training. Some midwives elaborated that some lecturers were using SBE during the training as a pedagogical method of training in healthcare. However, there were inadequate sessions related to SBE in classroom teaching, as well as simulation laboratory and clinical settings. Graduates stated that they had had insufficient sessions using the SBE approach as a pedagogical method of learning and teaching. Mostly, the students who had experienced these were not trained in how to use SBE.

*“…, but not all that know, everyone has learned a hard way on how to teach this course, and it involves teaching skills to students …, but there is no training that is designed to teach teachers how to use simulation-based learning to teach students, …” (Graduate 08)*

2. Sub-theme: Approach to learning and teaching

Various methods of teaching were mentioned by the informants, like bedside teaching, small or large group discussions, demonstrations, presentations, assignments, logbooks, blended learning, seminars, tutorials, real practices, and simulation. Most informants acknowledged that there are differences between SBE approaches and other pedagogic methods, though teachers were knowledgeable and competent in using other pedagogical approaches rather than SBE.

*“… Before they come to practical issues, they stay in class and use lectures, small or large group discussion, which is the main, but also, they use demonstrations to show what is being done. But also, they use bedside teaching when in the ward during rounds … (Graduate 05)”*

Informants further stated that some lecturers’ under-utilized simulation laboratories and learners were exposed to the patients without much practice in the simulation laboratory.

*“The difference is very big, even in my experience I remember when I was in the first year, our skills lab was not yet, yet it was not very good, so most of the time after the theory we go straight to the patient … I remember the hardship I experienced with the patient for the first time …, different from the current students that we teach theory and take them to the simulation lab, and then they go to the patient …, we see where the difference is in working and confidence and understanding …, they benefit a lot different from us who never managed to get through simulation.” (Graduate 11)*

3. Sub-theme: Learning practice in a clinical setting as opposed to the SBE approach

Some participants said that with crowds of students, and with an inadequate number of patients in clinical practice, the SBE approach gives the opportunity to all learners to acquire the intended competencies in a simulation laboratory, both technical and non-technical skills. This implies that clinical placement cannot ensure the clinical learning that the students need to become highly qualified healthcare workers while facing an inadequate number of patients.

*“It is advantageous because, considering the real clinical environment, you find that most of the time one patient is surrounded by around 10 students, … these students could be from different programs or Universities, so finding students who have learned is difficult, …, so students could not learn” (Lecturer 10)*

Some informants felt that the SBE approach is favorable and easy to practice, especially for sensitive procedures, as opposed to practicing with real patients, where students cannot practice several times. Some informants perceived the SBE approach as providing a good opportunity to grasp skills among learners, especially for larger groups of students. Also, some participants said that it is not possible for a large group of students to get the opportunity for each student to practice with the same patient, but with simulation, they get a chance to repeat the same procedures till they grasp the skills.

*“… for example, if you are teaching per vaginal examination (PV), … you cannot teach using one patient, … you must have dolls, test all of them, … and after everyone continues with his/her patient” (Lecturer 10).*

#### 3.2.2. Theme: Challenges in the Implementation of Simulation-Based Education

In this context, challenges refer to the situation where the use of SBE was impeded by various factors that obstructed the implementation of this approach. These include infrastructure and non-human resources; a pedagogical basis for SBE—the case of curriculum and guidelines; a lack of formal simulation training for educators; and a lack of implementation due to lack of skills.

1. Sub-theme: Infrastructure and non-human resources

Most of the informants reported that SBE implementation has been a challenging process due to inadequate resources. Some informants said that the simulation laboratory is very small compared to the number of students.

*“… my opinion first infrastructure is not enough, … that skills lab is not enough there is only one that is used by the whole University, … it would be increased in size and have partitions, … a challenge currently is that you will go and find a person from medicine using, … he/she will have to finish first then you get in after, … equipment are not well organized, … you can arrange them well, another person comes and disorganizes them, … takes them and leaves them unarranged as you had done … (Graduates 11).*

*“Space is not enough, as I have said, there is only one that should be added/expanded” (Graduates 11)*

Moreover, most informants had concerns about the lack of both consumable and non-consumable resources.

*“…, because at times you must get to your pocket to buy some items …, let’s say magnesium sulfate, sometimes you find they are not okay, and a student must use them … For example, you find they need a syringe 20cc, others are there, but 20cc that is a must is not there … Coming to the family planning side may be one of them is not there so you have to buy and if you buy you find that there are may be 20 students …, so you cannot buy all 20, you buy like two …, they use and repack and the other one has to use as a new one, … that is why I said there is still a shortage” (Graduates 09)*

*“For us here in the clinical area … there are places called preceptor corners we wish to have the equipment, and we don’t have them, but we do borrow …, there is a doll to demonstrate assistance to a baby to breathe “HELP BABY BREATH” … (Lecturer 04).*

2. Sub-theme: Pedagogic basis for SBE—the case of curriculum and guidelines

Most informants had major concerns about the curriculum used during their training, both knowledge-based and competence-based curricula, as there was no content related to SBE. It was reported that the curriculum did not mention anything about SBE throughout the training of nurses for undergraduate students or postgraduate programs. Moreover, some informants said that this could be given little attention in gaining knowledge and skills related to SBE. Furthermore, some of the informants spoke of the need for guidelines on how to use SBE in their teaching to facilitate self-directed learning in the simulation laboratory to ensure appropriate practice during the learning process. Participants urged mostly that there should be some checklists and scenarios. Some of the informants reported that no guidelines exist to guide learners and facilitators during practice.

*“… I have not seen a guideline on my side, but we learn using the available checklists, how to insert IUCD, … what are the steps, … checklist, we have them but not a general simulation, … We don’t have one … (Graduates 09).*

*“… Yes, there must be a guideline so that teaching is systematic… (Lecturer, 01)*

3. Sub-theme: Lack of implementation due to lack of skills and formal simulation training for educators

Informants also said that even if guidelines could exist, another pitfall is that most trainers are not competent in facilitating learning through a simulation-based education approach. Some of the informants said that different arenas could have been adequate for SBE, but none of them, in the university nor the clinic, is equipped even for basic skills training for SBE, and thus SBE becomes a challenge to implement.

*“Okay, I think trainers are a challenge because not every teacher has been trained on how to use SBE, so we are learning the hard way, there is no training that is designed to teach teachers how to use SBE, so I think even if we have a simulation lab that is equipped, we still want a teacher to be trained on how to use those simulations (Graduate 08)”*

*“A challenge is on trainers because not every lecturer has been trained on how to use simulation-based education, no pieces of training are designed to teach lecturers how to use simulation-based learning to teach students, so I think even if we have equipped simulation lab we still need lecturers to be trained how to use simulation in teaching” (Lecturer 01)*

#### 3.2.3. Theme—Ensuring Patients’ Safety

This theme critically describes how the SBE promotes patient safety in clinical settings. Since learners practice several times in the skill laboratory before being exposed to patients, it ensures minimal errors as learners are confident and competent when performing procedures on patients. Moreover, on-the-job training utilizing SBE for novice employees and students enhances the improvement of skills in clinical settings among employees as well as students.

1. Sub-theme: Builds student confidence

Most of the informants explained that SBE is a very efficient approach when it is well organized both in the simulation laboratory and clinical setting. Some informants elaborated that simulation laboratories give confidence as they practice more and when they go to the clinical setting, they are competent to perform procedures.

*“Okay, first it gives you confidence …; to do simulation I have a chance to repeat and repeat till I am confident …, I have confidence with the skill that I have practiced and mastered …, so I can provide service, … even ability to perform a procedure … I can perform it to the high level because I am confident … I know it” (Graduate 08)*

Moreover, some informants said that staff in the clinical area use SBE with students and new employees when they observe that they have skill deficits and thus practice in the preceptor corner to enhance novice skills.

*“… As an experienced midwife, a simulation-based practice we have even within my hospital …, we have those models and you can create a scenario and practice …, people can be able to gain competencies …, so it’s easy to create simulation and the people to get ideas in concerned topics …, then increase their knowledge and skills … (Graduate 12)*

2. Sub-theme: Creates a continuous practice environment

This describes the learning environment that allows students or novices to practice a certain procedure several times to gain the intended competencies. One informant says he used to see if there was a new technique or procedure introduced in the clinical area; they would go to the simulation corner within the hospital and simulate it. For participants, SBE is very useful to introduce new procedures in a simulated setting before implementing them in the clinical setting to patients. The staff in clinical settings get the opportunity to practice in the simulation lab or corners, and once they obtain competence, it is easy for them to implement. Not only that, but SBE is useful for on-the-job training at the workplace for new staff and seniors to improve care, as they said.

*“… At workplaces, there are these clinical instructors or mentors, they are there to help …, or if they are teaching junior staff or senior or if they want to improve something they can use that SBE approach … (Graduate 05)*

3. Sub-theme: Impact of SBE

Most of the informants perceived that the use of SBE had advantages for patients, lecturers, and learners. Some informants highlighted that SBE promotes patients’ safety as they will receive appropriate care from a skilled and competent provider. Also, it makes students and lecturers more confident as they gain more skills at the simulation laboratory before going to the real patients. Furthermore, the informants expressed their perception that SBE promoted patients’ safety and improved the quality of care for patients.

*“A big advantage is to help students build confidence; they know what I should do for their patient before I go into detail on the complaints that he/she has. It adds safety to the patient different from a student who comes from theory in class and goes directly to the patient, if a mistake is made one can cause danger to the patient, so if one has simulated, practiced, and is competent even if he/she goes to provide care to the patient, a patient is safer (Graduate 08)”*

*“A patient will receive care that is appropriate from a competent provider, so if one uses simulation and builds confidence, it means as they go to the clinical and continue to practice, he/she become competent, so a patient will receive competent-based care (Graduate 10)”*

Moreover, most informants stated that SBE is important for the lecturers to improve their skills and critical thinking to demonstrate certain procedures before even engaging learners in the simulation laboratory and clinical practice.

## 4. Discussion

The study aimed to explore perceptions and experiences of nurse educators (lecturers) and midwives on simulation-based education in Tanzania. Three themes emerged: (1) knowledge and skills on SBE, (2) challenges in the implementation of SBE, and (3) ensuring patients’ safety. The findings revealed that participants perceived that they were not trained on SBE, and nurse educators lack formal training to facilitate this approach. Despite the approach building confidence, as it allows continuous practice, participants experienced high numbers of students with inadequate infrastructure and resources for SBE as a major challenge.

### 4.1. Knowledge and Skills in Simulation-Based Education

The lecturers and graduates were aware of simulation-based education [39,40,41]; however, they had inadequate skills and lacked formal training on SBE. This might be a contributing factor for poor implementation of SBE in the institutions and is used occasionally to deliver knowledge and skills to learners during the learning process [25,42]. Training on simulation-based education in low-resource settings is very limited; most graduates learn theoretically rather than practicing [43]. However, the lack of training of SBE among lecturers is an indication that the approach had minimal support across the institution, and thus, the approach is not given priority. This could demoralize learners and lecturers from using this approach, as there is no role model or motivating environment for SBE.

The equipment available includes low-fidelity models [40] which enhance the technical and non-technical practice of learners, such as communication skills, teamwork, and decision-making [44]. However, the institutions also need to consider high-fidelity models, enhancing realism to promote critical thinking among lecturers and graduates. Some lecturers and mentors had a deficit in knowledge when it came to using the available simulators in the hospital preceptor corners, which demands training to facilitate frequent use of SBE for skills practice and transference into the clinical setting. However, the organization of the SBE laboratory is inadequate in low-resourced countries. Learners feel that the SBE laboratory is used when the need arises rather than a mandatory approach, which gives realism to their learning approach before being exposed to real patients in the clinical setting [17]. The organization of the SBE laboratory is an important factor for learners as it acts as a motivating environment for learning and to acquire the intended competencies. Institutions are urged to provide conducive environments for the theoretical and practical context for production of a skilled workforce.

### 4.2. Challenges in the Implementation of Simulation-Based Education

Institutions are facing inadequate infrastructure and non-human resources [45], which hinders the effective implementation of SBE. These limit SBE practice both in the simulation laboratory and clinical settings for learners to acquire the intended competencies [46,47]. Moreover, the SBE laboratory rooms are very small and hamper learners’ self-directed learning in the skill laboratory to practice learned skills several times for mastering [25,46]. Similarly, the implementation varied in each institution as there are no guidelines or policies [48] that harmonize with practice. This increases complexity, even making it very difficult for self-reflection learning during general practice for lecturers and self-directed learning among learners. Harmonized policies and guidelines are beneficial when peer learning in SBE is taking place to guide learners. The lecturers are struggling in their own way with how to transfer knowledge and skills using the SBE approach [25,49]. For effective practice, competent lecturers are the key to success for simulation-based education implementation in simulation laboratories and clinical settings across institutions. A lack of theoretical background among clinical instructors on the SBE approach limits their ability to assist learners or new employees [25] in the clinical setting. They fail to perform certain procedures, exposing patients to poor-quality care due to lack of skills [50], and learners sometimes are exposed to patients [50,51] without much practice in the preceptor corners or simulation laboratory. This highlights that there is little communication and even less dissemination of this approach, as our interpretation directly reveals that the SBE approach is given little attention. Neither undergraduate nor postgraduate curricula include any SBE content [51], which restricts students’ amount of practice upon graduating, as SBE was given less weight during their training despite being an important approach in learning. Higher learning institutions need to integrate simulation-based education (SBE) as a learning approach to enhance practice before learners are exposed to clinical settings, to promote patient safety, especially in Sub-Saharan Africa (SSA). The existing curriculum for the Master’s program mentions simulation only as a teaching strategy with limited utilization, and no simulation content was included in the undergraduate program.

### 4.3. Ensuring Patients’ Safety

Participants were knowledgeable about the potential impacts of the SBE approach on learners, lecturers, and patients. In the SBE approach, learners had a high chance of repeated practice to acquire the intended knowledge and skill as opposed to the traditional model of the lecturing-and-demonstrating approach. Learners were sometimes exposed directly to patients without much practice in the simulation laboratory, which might facilitate novice errors and promote patients’ insecurity [52,53]. However, if SBE is practiced effectively, it promotes patients’ safety and enhances patients’ quality of life as it is safe and with minimal errors, and it is free from incidental injuries from the learners [40,41,54,55,56]. Simulation is a well-established and effective learning method to protect patients from learner or novice errors. However, simulation training requires trained facilitators, particularly to lead the structured conversation known as debriefing after the simulation session. During debriefing, the experiences from the simulation are explored, analyzed, and synthesized to enhance knowledge and improve both clinical and non-technical skills, ultimately aiming to provide better healthcare. Peer-to-peer learning, or peer-assisted learning (PAL), where students learn together and from each other, has also been proven to be an effective learning method. This approach also has the potential to address the challenge of high student-to-teacher ratios in health education, which is the case in this setting.

Informants perceived that SBE could make a valuable contribution to the nursing curriculum and from there to patient care and safety [25]. There is an under-utilization of SBE, which necessitates students being exposed to practice with real patients with minimal practice in a simulation laboratory [25]. The use of SBE enhances confidence in practicing sensitive procedures rather than using real patients [41,53,56]. Practice with real patients has limitations, which also could cause trauma to patients, as opposed to using the SBE approach as a pedagogical method, which is free from harm and can be practiced more than once [25,40,42,53,56].

### 4.4. Strengths and Limitations

The data were collected in two high-learning institutions and their respective teaching hospitals, where participants had varied perceptions and experiences with SBE. Moreover, participants had a clear picture of their working environment. This enhances quality data collection for the study. However, the study inclusion criteria comprised all participants regardless of the year of graduation. This might have caused recall bias for some of the information from the participants, since it was very difficult to recall the whole scenario of training where they were trained. However, the interviewer used the same environment to conduct the interview, which enhanced confidence and trust among the participants to share their perceptions and experiences on the SBE approach. The study also collected data from two higher learning institutions, which could limit transferability. Moreover, the study reflects experiences at a single point and therefore cannot capture changes in long-term effects of simulation-based education.

To avoid institutional affiliation bias, all information identifying an institution, such as logos, was removed, and the same data collection tool was used for all participants. Moreover, social desirability bias was minimized by building trust between the interviewer and interviewee. The interviewer also maintained a non-judgmental approach and ensured anonymity during the interview process. A shared cultural background between the interviewer and interviewee, along with different disciplinary backgrounds, helped reduce power dynamics during the interview, and reflexivity minimized researcher influence during the interview process.

## 5. Conclusions

Midwife graduates perceived that they were not trained on the use of SBE, and nurse educators had no formal training on this approach. Participants noted a high number of students with inadequate infrastructure and resources, both in the simulation laboratory and clinical settings, as a major challenge. Policy and guidelines to practice an effective SBE approach are missing for nurse educators and learners for effective guidance and for self-directed learning or peer-to-peer learning. Surprisingly, both undergraduate and postgraduate curricula are missing SBE content. Therefore, simulation-based education is at an early stage of adoption in higher learning institutions in Tanzania and will require ongoing development, support and resources to fulfil its potential in promoting patient safety.

## Figures and Tables

**Table 1:** Participants’ sociodemographic characteristics n = 20.

Resp No.	Sex	Age	Education Level	Marital Status	Training Institution	Duration MSc Training (yrs)	Heard SBE	SBE Source of Information	Work Experience (yrs)
P1	F	40	MSc Midwifery	Married	MUHAS	2	Yes	School	13
P2	F	39	MSc Midwifery	Married	MUHAS	2	Yes	Teacher in class	16
P3	F	40	MSc Midwifery	Married	MUHAS	2	Yes	MSc school	7
P4	F	59	MSc Midwifery	Single	MUHAS	2	Yes	School	24
P5	F	55	MSc Midwifery	Married	MUHAS	2	No	Not heard	24
P6	F	42	MSc Midwifery	Married	MUHAS	2	Yes	School	5
P7	F	52	MSc Midwifery	married	MUHAS	2	Yes	School	17
P8	M	31	MSc Midwifery	Married	MUHAS	2	Yes	Class	4
P9	F	40	MSc Midwifery PhD	Married	Makerere University	2	Yes	Curricular	8
P10	F	38	MSc Midwifery PhD	Married	Makerere University	2	Yes	School	12
P11	M	31	MSc Midwifery	Single	China	2	Yes	School	4
P12	F	38	MSc Midwifery	Married	MUHAS	2	Yes	School	17
P13	F	46	MSc	Single	KCMUCo	2	Not heard	School	6
P14	F	46	MSc Midwifery	Married	KCMUCo	2	Yes	School	22
P15	F	62	MSc Midwifery	Married	KCMUCo	2	Yes	KCMC	22
P16	F	48	MSc Midwifery	Married	KCMUCo	2	Yes	KCMUCo	22
P17	F	32	Midwifery	Married	KCMUCo	2	No	No	3
P18	F	46	MSc Midwifery	Married	MUHAS	2	Yes	School/KCRI	20
P19	F	45	MSc Midwifery	Married	UDOM	2	No	No	20
P20	F	46	MSc Midwifery	Married	KCMUCo	2	Yes	School	15

**Table 2:** Composed themes and sub-themes.

Themes	Sub-Themes
Knowledge and skills in SBE	Lecturers and midwife graduates’ awareness of SBE Approach to learning and teaching Learning practice in a clinical setting as opposed to the SBE approach
Challenges in the implementation of SBE	Infrastructure and non-human resources Pedagogic basis for SBE—the case of curriculum and guideline. Lack of implementation due to lack of skills and formal simulation training for educators
Ensuring patients’ safety	Builds student confidence Creates a continuous practice environment Impacts of SBE

## Data Availability

The data presented in this study are available from the corresponding author upon reasonable request. The data are not publicly available due to privacy restrictions.
